# Magnetic Resonance guided Focused Ultrasound (MRgFUS) treatment of primary pancreatic and hepatic cancer: preliminary experience in tumor control

**DOI:** 10.1186/2050-5736-2-S1-A19

**Published:** 2014-12-10

**Authors:** Michele Anzidei, Alessandro Napoli, Beatrice Cavallo Marincola, Fulvio Zaccagna, Federica Ciolina, Giulia Brachetti, Mario Bezzi, Carlo Catalano

**Affiliations:** 1 Department of Radiological, Oncological and Anatomopathological Sciences, Sapienza University of Rome, Italy

## Purpose

To evaluate the feasibility of MRgFUS ablation in selected pancreatic and hepatic primary tumors.

## Materials and methods

After giving their informed consent 5 patients with histologically proven unresectable pancreatic adenocarcinoma and 1 patient with unresectable right lobe HCC (4 males, 2 females; age range 58-72) underwent MRgFUS treatment on a dedicated 3T unit featuring the ExAblate 2100 system (InSightec). The system is composed by a 200-element transducer located within the MR table. The MR guidance allows a detailed depiction and visualization of the lesion; moreover, the use of the proton resonance frequency (PRF) shift method allows a real time monitoring of the temperature inside the target lesion and the adjacent anatomical structures, in order to ensure adequate tissue ablation and safe ablation margins. The treatment was performed in general anesthesia with breath control. After the procedure, gadolinium-enhanced gradient echo T1-weighted sequences were performed in order to evaluate the ablated area and the absence of possible local complications. Clinical and imaging follow-up was performed with both MR and CT at 3 and 6 months after treatment respectively for the patient with HCC and those with pancreatic cancer.

## Results

Treatment was successfully performed in all patients without any adverse events during or after the procedure. MR images acquired immediately after treatment demonstrated necrosis of ablated area within the lesion in all cases; in particular the HCC was completely non-enhancing. At short term clinical follow-up, all the patients with pancreatic cancer referred reduction of pain symptoms due to infiltration of the celiac plexus. However follow-up imaging demonstrated recurrence of pathologic tissue within the ablated area, even if there was no local progression of the disease. Two patients with pancreatic cancer underwent radiotherapy after treatment, while the remaining one underwent another MRgFUS ablation.

## Conclusions

Our preliminary clinical experience suggests that MRgFUS is a feasible and repeatable ablative technique in patients with unresectable and device-accessible hepatic and pancreatic lesions.

